# Factors affecting formula compliance of infants with IgE mediated cow's milk protein allergy during the pandemic

**DOI:** 10.3389/falgy.2023.1017155

**Published:** 2023-05-05

**Authors:** Dilara Kocacik Uygun, Betul Karaatmaca, Erdem Topal, Mustafa Arga, Ozlem Sancakli, Dilek Ozcan, Mahir Igde, Sukru Cekic, Gulbin Bingol, Betul Buyuktiryaki, Cansin Sackesen, Alihan Sursal, Fatih Ozdener, Aysen Bingol

**Affiliations:** ^1^ Department of Pediatric Allergy-Immunology, Akdeniz University School of Medicine, Antalya, Turkey; ^2^ Department of Pediatric Allergy and Immunology, University of Health Sciences, Ankara Bilkent City Hospital, Ankara, Türkiye; ^3^ Department of Pediatric Allergy-Immunology, Inonu University Faculty of Medicine, Turgut Ozal Medical Center, Malatya, Turkey; ^4^ Department of Pediatric Allergy and Immunology, Istanbul Medeniyet University Faculty of Medicine, Göztepe Prof Dr Süleyman Yalçın City Hospital, Istanbul, Türkiye; ^5^ Department of Pediatric Allergy and Immunology, Zübeyde Hanım Practice and Research Center, Baskent University, İzmir, Türkiye; ^6^ Department of Pediatric Allergy-Immunology, Cukurova University School of Medicine, Adana, Turkey; ^7^ Department of Pediatric Allergy-Immunology, Istinye University Liv Hospital, Istanbul, Turkey; ^8^ Department of Pediatric Allergy-Immunology, Uludag University School of Medicine, Bursa, Turkey; ^9^ Department of Pediatric Allergy and Immunology, Acıbadem Mehmet Ali Aydınlar University, Istanbul, Türkiye; ^10^ Department of Pediatric Allergy, Koc University School of Medicine, Istanbul, Turkey; ^11^ Department of Neuroscience, Bahcesehir University School of Medicine, Istanbul, Turkey; ^12^ Department of Pharmacology, Bahcesehir University School of Medicine, Istanbul, Turkey

**Keywords:** treatment adherence, CMPA, IgE mediated food allergy, infants, COVID-19 pandemic

## Abstract

**Introduction:**

Cow's milk protein allergy (CMPA) is the most commonly encountered food allergy in the world, usually seen in infants under the age of 2 years. This study aims to determine the factors including COVID-19 affecting formula compliance of CMPA patients.

**Methods:**

This study is a prospective, observational study based on 10 different Paediatric Allergy-Immunology clinics in Turkey. Patients aged between 6 months and 2 years, who were followed up with IgE-mediated CMPA treatment or newly diagnosed and using breast milk and/or formula were included in the study. The sociodemographic characteristics of the patients, their symptoms, the treatments they received, and the effects of the COVID-19 pandemic on adherence to formula were evaluated with a questionnaire administered to the parents.

**Results:**

The compliance rate for formula-based treatment was 30.8% (IQR: 28.3, SD: 21.86). The number of patients with a single and multiple food allergy was 127 (51.6%) and 71 (28.9%), respectively. Breastfeeding duration, daily amount of prescribed formula and addition of sweetener to the formula were found to reduce compliance (*p* = 0.010, *p* = 0.003, and *p* = 0.004, respectively). However, it was determined that the patient's height, weight, age at diagnosis, and age of formula onset did not have a significant effect on compliance.

**Conclusion:**

It was found that the duration of breastfeeding, the increase in the daily amount of formula requirement, and the addition of sweeteners had adverse effects on formula compliance. There was no significant correlation between the formula adherence of CMPA patients and the pandemic.

## Introduction

Cow's milk protein allergy (CMPA) is the most common food allergy. It is usually seen in infants and children under 2 years of age and is often formed by immunoglobulin E (IgE) (1). Foods containing cow's milk are the basic elements of infant nutrition. In its treatment, mother and baby should follow a cow's milk protein diet. Special formulas are used in cases where breast milk is not available and/or insufficient (2–5). One of the most important factors affecting the good clinical outcome in treatment is compliance with treatment. Compliance can be affected by a variety of reasons (6, 7). With the onset of the COVID-19 pandemic, the decrease in outpatient applications for various reasons has adversely affected the treatment processes of all allergic patients.

This study aims to determine the formula-based compliance with CMPA treatment and to determine the main factors affecting formula compliance during the COVID-19 pandemic of CMPA patients admitted to the hospital in Turkey. Moreover, it aims to evaluate the demographic and anthropometrical characteristics as well as other types of food allergy distribution of these patients.

## Methods

Our study is a multicentre, prospective, observational study and 10 different Paediatric Allergy-Immunology clinics from Turkey participated. Patients aged between 6 months and 2 years who were followed up with IgE-mediated CMPA or newly diagnosed and using breast milk and/or formula were included. Diagnosis of IgE-mediated CMPA were made by sudden hypersensitivity reaction symptoms after exposure to allergens (urticaria, angioedema, oral allergy syndrome, wheezing, rhinitis, anaphylaxis etc.) and specific IgE (sIgE > 0.35 kU/L) elevation and/or skin prick test or with the prick-to-prick test (≥3 mm from the negative control).

Our survey includes only hypoallergenic formulas including amino acid based, extensively hydrolysed and rice-based formulas. Two different surveys were conducted to patients, “survey questions about the COVID-19 Pandemic” and “factors affecting adherence to formula-based CMPA treatment”, respectively. Questionnaires were conducted by Pediatric Allergy-Immunology physicians as face-to-face and/or by telephone interviews to evaluate the sociodemographic characteristics of the patients, their symptoms, the treatments they received, and the effects of the COVID-19 pandemic on their adherence to formula.

Two different adherence percentages were calculated. Whole group formula-based adherence and non-breastfed group formula-based adherence act as the dependent variable during regression analysis. The percentage of patients’ compliance with formula was found by dividing the amount of formula that should be prescribed daily by the amount of formula consumed by the patients in a day aside from breastfeeding adherence and supplemental elimination diet adherence. For few infants between 2 and 6 months of age, mother diet adherence was calculated for minimum daily breastfeeding for every age period coherent with WHO and UNICEF recommendations on breastfeeding, which is exclusive breastfed for the first 6 months (8–12 times daily). The amount of consumed supplementary elimination diet outside of hospital is unknown.

Cases that were not given informed consent by their parents, did not complete the survey questions, and did not attend their follow-up regularly were excluded from the study.

### Statistical analysis

Statistical tests and analyses were performed using IBM SPSS Statistics for Windows v20.0 software (IBM Corp., Armonk, NY, US). The normality of the distribution of continuous variables was determined using the Shapiro–Wilk test. Chi-square test was used for categorical variables, and categorical distributions were expressed as number of samples and percentage. Chi-square test was performed to cross-tabulate data sets with more than 5 samples and examine the categorical distribution. Fisher's Exact test was performed to analyse data sets with less than five samples. The Mann–Whitney *U* test was performed to examine the distribution of the independent continuous variable that did not show normal distribution according to the binary categorical variable, and the Kruskall–Wallis test was performed to examine the distribution according to more than 2 categorical variables. The relationship between the continuous dependent variable and more than one independent variable was analysed with multiple linear regression and the relationship between a single independent variable with simple linear regression. The standard beta weights of the results that were significant in the linear regression were multiplied by the standard deviation of the dependent variable, and the effect of an increase as much as the standard deviation of the independent variable on the dependent variable was calculated. During stepwise multiple regression modelling, independent variable selection was achieved by applying Pearson correlation to all possible variables based on the dependent variable. The multiple regression was modelled step by step, starting with the independent variable showing the highest correlation with the dependent variable in the Pearson correlation matrix, and the modelling was stopped at the first statistical insignificance, and the confidence interval level was determined as 95%. When the percent difference between the B coefficients in the multiple linear regression results of the same independent variable was 10 or more, they were considered to be confounders with another independent variable. The tolerance threshold for the presence of collinearity was accepted as 0.200. The relationship between the categorical dependent variable and the independent variables and their odds ratio (OR) were analysed by multiple logistic regression. The risk ratios (RR) of the variables were calculated using the log-binomial generalized linear model. Statistical significance level was accepted as *p* < 0.05.

### Ethical approval

The study was approved by the Akdeniz University Faculty of Medicine Ethics Committee [decision no. 70904504/585, dated 10.09.2020]. Moreover, all participants provided informed consent in the format required by the relevant authorities and/or boards.

## Results

246 patients (97 [39.4%] female and 149 [60.6%] male) with a mean age of 11.8 ± 3.02 (IQR: 3.5) months were included. Sociodemographic characteristics of the patients and their statistical analysis are given in Tables 1A, B, respectively. The compliance rate for overall formula-based treatment was calculated as 30.8% (IQR: 28.3, SD: 21.86) and non-breastfeeding formula-based treatment as 38.3% (IQR: 32.1, SD: 32.7). 7 (2.8%) patients in the study were diagnosed with COVID-19. Survey details are given in Supplementary Table S1 and S2.

**Table 1A:** Patient demographics.

Demographics	N (%)
Sex, *n* (%)	
Boys	149 (60.6)
Girls	97 (39.4)
Age, mean ± SD (IQR), month	11.8 ± 3.02 (9.5–13.0)
Age at symptom onset, mean ± SD (IQR), month	3.6 ± 2.8 (2.0–5.0)
Age at diagnosis, mean ± SD (IQR), month	5.3 ± 3.2 (3.0–6.0)
Breastfeeding duration, mean ± SD (IQR), month	10.4 ± 7.4 (6.0–14.0)
Formula starting age, mean ± SD (IQR), month	5.7 ± 3.1 (4.0–7.0)
Formula Type, *n* (%)	
Amino acid-based formula	192 (78.1)
Extensively hydrolysed formula	24 (9.8)
Riced-based formula	8 (3.3)
Formula usage duration, mean ± SD (IQR), month	8.1 ± 6.7 (3.0–12.0)
Required amount of daily formula intake, mean ± SD (IQR), ml	1,153.6 ± 268.13 (997.8–1,330.4)
Whole group formula adherence, mean ± SD (IQR), %	30.8 ± 21.9 (11.08–39.38)
No breastfed formula adherence, mean ± SD (IQR), %	38.3 ± 37.5 (14.70–46.61)
2–6-month age mother diet adherence, mean ± SD (IQR), %	27.7 ± 38.4 (0–37.2)
2–6-month age formula adherence, mean ± SD (IQR), %	41.8 ± 21.6 (17.3–32.0)
Birth Weight, mean ± SD (IQR), g	3,200.0 ± 641.8 (2,900.0–3,500.0)
Current Height, mean ± SD (IQR), cm	79.4 ± 61.1 (70.0–82.0)
Current Weight, mean ± SD (IQR), g	6,985.3 ± 4,649.4 (1,225.0–9,900.0)
Head Circumference, mean ± SD (IQR), cm	45.6 ± 2.9 (44.0–48.0)
Maternal Age, mean ± SD (IQR), year	30.2 ± 5.0 (27.0–33.0)
Paternal Age, mean ± SD (IQR), year	33.9 ± 5.4 (33.0–37.0)
COVID-19 Diagnosis, *n* (%)	7 (2.8)

SD, standard deviation; IQR, interquartile range.

As expected, patient age is directly proportional with the breastfeeding duration (*p* < 0.001). Required amount of daily formula intake showed inverse correlation with duration of breastfeeding (*p* = 0.004, −0.202) and direct correlation with adherence (*p* < 0.001, 0.922). Adherence was slightly higher in the case of early formula starters (*p* = 0.003, −0.230) and patients with lower weight scores at CMPA diagnosis (*p* = 0.045, −0.154). Formula type showed no correlation between adherence (*p* = 0.585). Addition of sweetener showed a positive correlation on duration of breastfeeding (*p* = 0.005) and a negative impact on the required amount of daily formula intake (*p* = 0.004). Different types of sweeteners of different formula types did not show any correlation with adherence (*p* = 0.223). Breastfeeding durations of the patients using extensively hydrolysed formula were shorter compared to patients using other formula types (*p* = 0.013). Moreover, patients using amino acid-based formula were older than patients using other types of formulae (*p* = 0.001). There was no correlation between formula type and the required amount of daily formula intake (*p* = 0.604) (Table 1B).

**Table 1B:** Statistical analysis with the conclusion parameters.

Correlation Parameters		*p-*value	Correlation Coefficient^a^ or mean rank difference (%)^b^
Age, month	Duration of breastfeeding, month	<0.001	0.467
Age, month	Formula-based treatment adherence	0.612	NA
Age, month	Required amount of daily formula intake, ml	0.360	NA
Required amount of daily formula intake, ml	Duration of breastfeeding, month	0.004	−0.202
Required amount of daily formula intake, ml	Formula-based treatment adherence	<0.001	0.922
Formula-based treatment adherence	Current Height, cm	0.414	NA
Formula-based treatment adherence	Current Weight, g	0.045	−0.154
Formula-based treatment adherence	Head Circumference, cm	0.487	NA
Formula-based treatment adherence	Formula starting age, month	0.003	−0.23
Is sweetener added to the formula? 1: no; 2: yes	Age, month	0.073	NA
Is sweetener added to the formula? 1: no; 2: yes	Duration of breastfeeding, month	0.005	19.0 (2 > 1)
Is sweetener added to the formula? 1: no; 2: yes	Required amount of daily formula intake, ml	0.004	21.3 (1 > 2)
Is sweetener added to the formula? 1: no; 2: yes	Formula Type (1, 2, 3)^c^	0.785	NA
Formula Type (1, 2, 3)^c^	Duration of breastfeeding, month	0.013	40.8 (1 and 3 > 2)
Formula Type (1, 2, 3)^c^	Age, month	0.001	38.6 (1 > 2 and 3)
Formula Type (1, 2, 3)^c^	Required amount of daily formula intake, ml	0.604	NA

^a^ For Pearson and Spearman's correlations.

^b^ For Mann–Whitney *U* test.

^c^ 1: Amino acid-based formula, 2: Extensively hydrolysed formula, 3: Riced-based formula.

**Figure 1:**
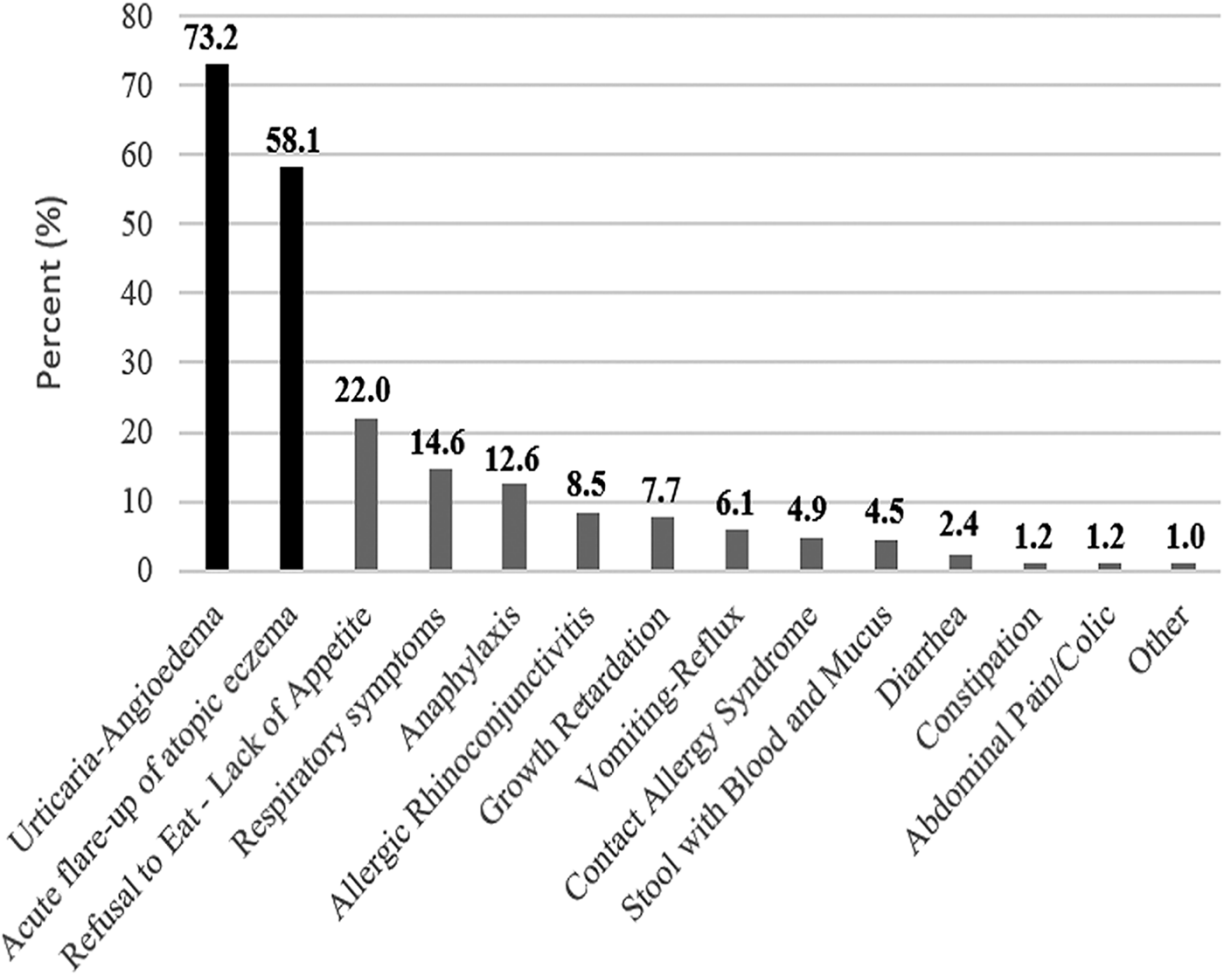
Overall food-related finding frequencies. Food-related symptom percentages of all patients diagnosed with CMPA were sorted from highest prevalence to lowest.

The number of patients with single food allergy was 127 (51.6%), and the number of patients with multiple food allergy was 71 (28.9%). Moreover, there was no any statistical difference (*p* = 0.535) in formula adherence between patients with single and multiple food allergy (Table 2). In addition to CMPA, the most frequently detected food allergy is egg (153 patients, 62.2%) (Supplementary Table S3). In addition to the clinical presence of CMPA, having egg, goat milk and veal allergies showed no difference in terms of adherence (*p* < 0.219). The most common finding after food exposure was urticarial-angioedema, and it was detected in 180 (73.2%) patients (Figure 1).

**Table 2:** Food allergy types and food-related symptoms accompanied with CMPA allergy according to 6–24 months.

Clinical characteristics	Total *n* (%)	*p*-value
FA type		
Single food	127 (51.6)	0.535
Multiple food	71 (28.9)	
Food-related findings		
Urticarial-Angioedema	180 (73.2)	
Acute flare-up of atopic eczema	143 (58.1)	
Refusal to Eat—Lack of Appetite	54 (22.0)	
Respiratory symptoms (wheezing, cough, dyspnoea)	36 (14.6)	
Anaphylaxis	31 (12.6)	
Allergic Rhino conjunctivitis	21 (8.5)	
Growth Retardation	19 (7.7)	
Vomiting-Reflux	15 (6.1)	
Contact Allergy Syndrome	12 (4.9)	
Stool with Blood and Mucus	11 (4.5)	
Diarrhoea	6 (2.4)	
Constipation	3 (1.2)	
Abdominal Pain/Colic	3 (1.2)	
Other	2 (1.0)	

FA, food allergy.

**Figure 2:**
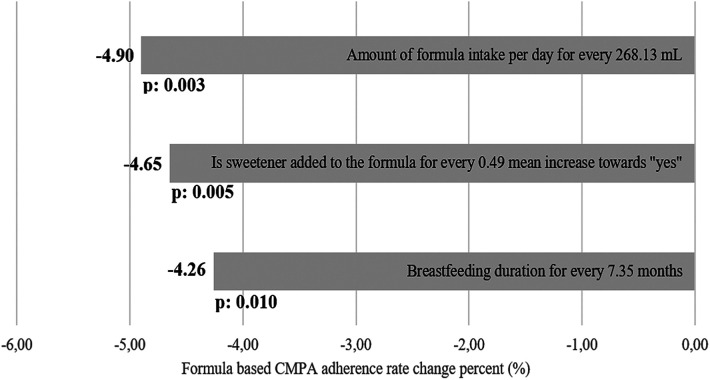
Beta weight estimation as overall percentage change of formula-based CMPA treatment adherence rate together with related *p*-values. For every SD of increase of the variable, a percentage difference of overall formula-based CMPA treatment adherence rate was estimated and sorted from highest to lowest.

The multiple linear regression analysis, which includes the factors affecting the rate of adherence to treatment and considering possible cofounder relationships, is given in Table 3A and comparatively demonstrated in Figure 2. The mean rate of compliance with CMPA treatment of 169 (68.7%) patients using formula was calculated as 30.8% (IQR: 28.3, SD: 21.86). It was observed that the compliance rate was reduced by the increased duration of breastfeeding (months), the amount of formula that should be consumed daily (ml), and by the addition of sweetener to the formula (*p* = 0.010, *p* = 0.003, and *p* = 0.004, respectively). In the evaluation of standard beta weights of the variables affecting adherence to CMPA treatment, it is seen that an increase of one standard deviation (7.35 months, 268.13 ml, and 49% increase in the mean of patients with sweetener added) causes a decrease of 4.26%, *p* = 0.01, 4.90%, *p* = 0.003, and 4.65%, *p* = 0.004%, respectively. Only formula intake per day showed positive correlation with formula specific adherence rate in non-breastfed patients as every 307.78 ml increase of the required amount of formula intake increase formula-based adherence by 3.08% (*p* < 0.001).

**Table 3A:** Multiple linear regression analysis for factors affecting CMPA treatment adherence.

Dependent Variable, unit mean (SD)	Independent Variable, unit mean (SD)	*B* coefficient	Cofounder, %	Multiple Regression Standardized Beta Weight	*p-*value	Standardized Beta Weight Interpretation^a^
Formula-specific CMPA adherence rate, % 30.8 (21.86)	Required amount of formula intake per day, ml	−0.019	Present, 21.1	−0.224	0.003	−4.90%
	1,153.6 (268.13)					
	Is sweetener added to the formula? 1: no; 2: yes	−9.277	Absent, 0.8	−0.213	0.004	−4.65%
	1.4 (0.49)					
	Breastfeeding duration, mo	−0.621	Present, 40.0	−0.195	0.010	−4.26%
	10.4 (7.35)					
Non-breastfed formula-specific CMPA adherence rate, % 38.3 ± 37.5	Required amount of formula intake per day, ml	0.082	NA	0.846	<0.001	3.08%
	1,206.5 (307.78)					
Required amount of daily formula intake, ml 1,153.6 (268.13)	Breastfeeding duration, mo	10.288	NA	0.249	<0.001	66.76 ml
	10.4 (7.35)					

SD, standard deviation; NA, not applicable.

^a^ Standardized beta weight interpretation on adherence rate for significant results per single SD increase of the independent variable.

In the multiple linear regression model, a cofounder relationship was observed between the duration of breast milk intake (months) and the amount of formula that should be consumed per day (ml) (40.0% and 21.1%, respectively). Thus, a simple linear regression was modelled by taking the amount of formula that should be consumed per day (ml) as the dependent variable and the duration of breast milk intake (months) as the independent variable was performed.

It was observed that each SD increase in the duration of breastfeeding and the amount of formula consumed daily were the variables with the highest inverse ratio with the rate of compliance with CMPA treatment. These results were also evaluated with multiple logistic regression analysis and the mean compliance rate was taken as 30% as the threshold value. It was observed that the duration of breast milk intake (months), the required amount of formula to be consumed daily (ml), and the addition of sweetener to the formula in the whole group and the daily required amount of formula provided significant correlations between patients whose compliance with CMPA treatment was less than 30% (*p* = 0.032, *p* = 0.030, *p* = 0.008, and 0.001 respectively). The distribution of patients in the treatment compliance subgroups (<30% or ≥30%) according to the duration of breast milk intake (months) and the required amount of formula that should be consumed per day (ml) is almost equal (β: −0.059, OR: 0.943, RR: 1.020 and β: −0.001, OR: 0.999, RR: 1.001, respectively). On the other hand, the probability ratio of patients who added sweetener to the formula to be less than 30% of formula compliance was observed as *B*: −0.937, OR: 0.392, RR: 1.335. Lastly, the probability ratio of non-breastfed patients to be less the 30% formula compliance was observed as *B*: −0.093, OR: 0.911, RR: 1.012 (Table 3B). Non-breastfed formula adherence rate was also inversely affected from the required amount of formula.

**Table 3B:** Multiple logistic regression analysis for factors affecting formula-based CMPA treatment adherence.

Dependent Variable (*n*, %)	Independent Variable, unit mean (SD)	*B* coefficient	Odds Ratio	*p* value	Risk Ratio
Formula-specific CMPA adherence rate <30% (101, 59.4) vs. ≥30% (69, 40.6)	Required amount of daily formula intake, ml	−0.001	0.999	0.030	1.001
	1,153.6 (268.13)				
	Is sweetener added to the formula? 1: no; 2: yes	−0.937	0.392	0.008	1.335
	1.4 (0.49)				
	Breastfeeding duration, mo	−0.059	0.943	0.032	1.020
	10.4 (7.35)				
Non-breastfed formula-specific CMPA adherence rate, % 38.3 ± 37.5	Required amount of formula intake per day, ml	−0.093	0.911	0.001	1.012
	1,206.5 (307.78)				

SD, standard deviation.

In the examination of the survey answers for the COVID-19 pandemic, it was seen that 43.1% of the patients had a decrease in their family income. When the compliance rates of the children of families with and without decreasing income were compared to CMPA treatment, no significant correlation was found with either the linear regression method (*p* = 0.793) and the Mann–Whitney *U* test (*p* = 0.645). Similarly, no significant results were detected in the rates of adherence to treatment in patients who could not come to the hospital for various reasons (*p* = 0.349, *p* = 0.831, respectively) and had difficulty in communicating with healthcare personnel (*p* = 0.533, *p* = 0.381, respectively) during the pandemic. There was also no significant difference between the compliance rate of the group stating that the pandemic affected CMPA treatment and the patient's nutrition, and the unaffected group (*p* = 0.917, *p* = 0.669, respectively).

## Discussion

CMPA is the most common food allergy in childhood. Compliance with CMPA treatment is critical for the growth and development of babies. Due to the decrease in hospital admissions of allergic children during the COVID-19 pandemic, it adversely affected the treatment processes. In our study, we aimed to determine the main factors affecting the adherence to treatment during the pandemic process of patients who were followed up and/or newly diagnosed with CMPA during the pandemic.

Direct correlation of daily formula intake with adherence was expected as the more of the required amount of daily formula intake, more of the chance to fulfill the daily requirement. Moreover, slightly higher adherence in early formula starters and lower weight scores may result in habit development towards formula usage and may result in a worse clinical status which may increase the motivation towards formula usage, respectively. The positive relationship between addition of sweetener to the formula and duration of breastfeeding is somewhat surprising and may be explained as the average age of sweetened formula users were younger than non-sweetened formula (17.2 months vs. 21.0 months), thus their breastfeeding durations were also lower. Sweeteners and aroma givers are rarely used in cases with taste problems. It is often discontinued after the period of getting used to the food. Mostly amino-acid based formula was used in the study but one patient was fed with non-hydrolyzed lactose-free formula as that patient was started with a normal lactose-free formula in an external center due to some suspected symptoms, including colic. After the diagnosis for CMP allergy, a new arrangement for the appropriate formula was made for that specific patient.

Although we expected to see a positive correlation between adherence and the required amount of daily formula intake, it was observed that the formula-based compliance rate was negatively affected by the duration of breastfeeding, the required amount of formula to be consumed daily, and the addition of sweetener to the formula. The negative effect of sweetener might be related to taste habits, e.g. disliking both the sweetened or unsweetened taste of the formula.

Formula-based adherence percentage of non-breastfed patients was not much higher (approximately 8%) compared to the whole group. The required amount of formula intake in non-breastfed infants showed a positive correlation with formula-based treatment adherence despite the negative correlation in the whole group. This may underline the awareness of the parents towards the need of formula adherence in the absence of breastfeeding, which opposed to whole group analysis that the required formula amount can be partially replaced with breastfeeding. In a similar study, the rate of compliance with formula-based treatment was found to be 65.7%, according to the opinions of paediatricians (7). However, the views of physicians were considered while calculating the rate in this study. The reason why the compliance rate was found to be 30.8% in our study resulted by calculating formula-based nutrition and lack of nutrition-based adherence outside of formula usage. In addition, the severity of the disease, the level of family education and the taste characteristics of the product were stated as the most affecting factors. In our study, it was found that the duration of breastfeeding, the increase in the required amount of daily formula uses and the addition of sweetener had adverse effects on compliance. This situation is similar to the study above. The amount of prescribed formula is calculated according to weight of the baby. As the baby grows, the proportion of formula that they receive decreases relatively, as they receive additional foods. It was also found that adding sweetener to the formula reduced compliance. Contrary to what is expected, the addition of sweetener does not increase compliance when the baby does not take the formula. Therefore, the baby should be allowed to adapt the formula without adding sweetener to it.

Surprisingly, there is no difference in terms of compliance rate between the patients who stated that the pandemic affected nutrition and those who stated that it did not. There was no difference between the two groups since the calculated compliance rate was limited by the use of formula, and it may change with increasing the number of patients and information on breastmilk/additional food intake. Or, it may be that the pandemic makes it difficult to comply with CMPA treatment, although it does not significantly impair compliance, and the decrease in income may act as problems that cause the family both to suffer from less income and to be exposed to stress due to difficulties in reaching the hospital and/or health personnel. The reason why there was no significant correlation between formula use and compliance rate during the COVID-19 pandemic may be due to the low number of patients whose use increased and decreased (8.1%). The decrease in the compliance rate of patients with an increase in appetite during the pandemic can be explained as a decrease in the use of formula due to the increase in the consumption of complementary foods or breast milk. However, since the number of patients with an increased appetite was significantly low (3.3%), the statistical power was insufficient and the analysis was insignificant even if the difference between the means of compliance of the patients whose appetite did not change and the patients with an increased appetite was high (29.9% vs. 16.3%) (*p* = 0.093 and *p* = 0.094, respectively). Similar studies published before the pandemic resulted much higher compliances to CMPA treatment compared to our study (85% and 65.7%, respectively) (7, 8). Considering that our study focuses on formula-based adherence in elimination diet and the lack of any relationship between pandemic and nutrition according to patients, the reason for our significantly lower adherence score seems to be due to our limitation of focusing on formula-based adherence without the addition of daily meals.

The decrease in the rate of adherence to treatment of patients whose CMPA symptoms decrease can be explained as a decrease in the severity of the disease. However, this situation was not detected in the regression analysis. The number of patients who had difficulty in finding a formula and whose treatment compliance rate was calculated was insufficient (1.6%), so it could not be analysed statistically.

While calculating the compliance rate to CMPA treatment, only the use of formula was taken into account. Since the supplementary food and breast milk used by the patients were not included in this calculation, it is thought that the compliance rate gave results below the true value. Moreover, the factors affecting the compliance were analysed over the formula-specific compliance ratio. Compliance rates of some patients to CMPA treatment could not be calculated due to data loss. This situation led to the inclusion of less than the number of patients included in the analysis, which examines the factors affecting compliance.

In conclusion, according to our study, there was no significant correlation between the COVID-19 pandemic and formula adherence of the patients. However, it was found that a longer duration of breastfeeding is inversely correlated with formula adherence. Similarly, the increase in the daily required amount of formula also showed inverse correlation with adherence in whole group while the opposite is true for non-breastfed patients. Lastly, the addition of sweeteners also had negative effects on formula compliance, which underlines how those patients have to be forced to get used to the sweetener-free formula.

### Study limitations

Our study lacks the knowledge of supplemental elimination diet adherence. Recommended daily formula intake was calculated aside from breastfeeding adherence and supplemental elimination diet adherence and “CMPA treatment adherence” was partially evaluated. This calculation causes our formula-based adherence to be low and push us to focus only to formula-based adherence part of the CMPA treatment adherence. Mother diet adherence were calculated for patients under 6 months of age who were prescribed for formula due to the requirement of exclusive breastfeeding in order to observe the factors affecting formula-based adherence by excluding supplementary elimination diet. However, the number of patients under 6 months of age who were prescribed for formula were not sufficient for any statistical analysis. Thus, we made a sub-group analysis on patients with zero mother diet adherence in addition to the whole group analysis to focus and compare the factors affecting formula-based adherence.

All patients were recorded during the pandemic; thus, our study lacks a control group consisting of patients without CMPA but with other food allergies to compare the effect of the clinical presence of CMPA on dietary adherence. As aforementioned, study also lacks a cohort recorded before the pandemic, which prevents a clear comparison between adherence during and before pandemic. To cover these limitations, similar study results were referred and compared with our data in the discussion section.

## Data Availability

The data analyzed in this study is subject to the following licenses/restrictions: The retrospective data used to support the findings of this study are restricted by the Bakirköy Sadi Konuk Research and Training Hospital Ethics Committee in order to protect patient privacy. Data are available for researchers who meet the criteria for access to confidential data.
