# Supplementary material for: Factors affecting formula compliance of infants with IgE mediated cow's milk protein allergy during the pandemic
Source: Front Allergy. 2023 May 5;4:1017155. doi: 10.3389/falgy.2023.1017155 (PMC10198129; doi:10.3389/falgy.2023.1017155)
Supplement: Supplementary file 1 [file Table1.docx]

**Supplementary Table 1.** Response percentages of survey questions about the COVID-19 Pandemic.

| **Working status of your parents during the COVID-19 pandemic** | | | | |  |  |
| --- | --- | --- | --- | --- | --- | --- |
| No Answer | Only the mother works | Only the father works | They both work | Unemployed |  |  |
| 0.4 % | 0.8 % | 73.2 % | 21.2 % | 4.5 % |  |  |
| **Has there been a decrease in your income level during the COVID-19 pandemic?** | | | | | | |
| No Answer | No | Yes |  |  |  |  |
| 0.8 % | 56.1 % | 43.1 % |  |  |  |  |
| **Have you visited a hospital during the COVID-19 period?** | | | | |  |  |
| No Answer | No | Yes |  |  |  |  |
| 0.8 % | 11.0 % | 88.2 % |  |  |  |  |
| **Have you ever had suspected COVID 19 symptoms and did not go to the hospital out of fear?** | | | | | | |
| No Answer | No | Yes |  |  |  |  |
| 0.8 % | 78.5 % | 20.7 % |  |  |  |  |
| **How was transportation to the hospital provided during the COVID-19 pandemic?** | | | | |  |  |
| No Answer | Private vehicle | Public transport | Ambulance | Walking |  |  |
| 4.1 % | 85.8 % | 9.8 % | 0.0 % | 0.4 % |  |  |
| **Were you able to communicate with the doctor during the COVID 19 period?** | | | | |  |  |
| No Answer | No | Yes |  |  |  |  |
| 4.9 % | 6.5 % | 88.6 % |  |  |  |  |
| **If yes, specify?** | | | | |  |  |
| No Answer | Email | Online | Whatsapp | Phone | Hospital | Clinic |
| 7.3 % | 5.3 % | < 0.1 % | 24.0 % | 39.4 % | 66.7 % | 6.5 % |
| **Are there any COVID-19 positive individuals around?** | | | | |  |  |
| No Answer | No | Yes |  |  |  |  |
| 13.4 % | 84.6 % | 2.0 % |  |  |  |  |
| **Has the baby been diagnosed with COVID19?** | | | | |  |  |
| No Answer | No | Yes |  |  |  |  |
| 0.0 % | 97.2 % | 2.8 % |  |  |  |  |
| **Has the COVID-19 pandemic process affected your child's CMPA treatment and nutrition?** | | | | | | |
| No Answer | No | Yes |  |  |  |  |
| 4.1 % | 85.4 % | 10.6 % |  |  |  |  |
| **Has there been any change in breast milk during the COVID-19 pandemic?** | | | | | | |
| No Answer | No | Increased | Decreased |  |  |  |
| 8.9 % | 67.1 % | 0.4 % | 23.6 % |  |  |  |
| **Have there been any changes regarding the use of formula during the COVID-19 pandemic?** | | | | | | |
| No Answer | Decreased a lot | Decreased | Did not change | Increased | Increased a lot |  |
| 6.5 % | 4.7 % | 7.8 % | 74.6 % | 11.6 % | 0.4 % |  |
| **Has your child's appetite changed during the COVID-19 pandemic?** | | | | | | |
| No Answer | No | Decreased | Increased |  |  |  |
| 0.4 % | 82.1 % | 13.4 % | 4.1 % |  |  |  |
| **Have you used additional vitamins during the COVID-19 pandemic?** | | | | |  |  |
| No Answer | No | Yes |  |  |  |  |
| 3.7 % | 65.4 % | 30.9 % |  |  |  |  |
| **Did the mother have to be separated from the child during the COVID-19 pandemic?** | | | | | | |
| No Answer | No | Yes |  |  |  |  |
| 2.8 % | 92.3 % | 4.9 % |  |  |  |  |
| **Has there been an increase in cleaning habits during the COVID-19 pandemic?** | | | | | | |
| No Answer | No | Yes |  |  |  |  |
| 0.8 % | 17.1 % | 82.1 % |  |  |  |  |
| **Have you had any trouble finding a formula during the COVID-19 pandemic?** | | | | | | |
| No Answer | No | Yes |  |  |  |  |
| 6.9 % | 89.0 % | 4.1 % |  |  |  |  |
| **Has the habit of eating at home increased during the COVID-19 pandemic?** | | | | | | |
| No Answer | No | Yes |  |  |  |  |
| 0.0 % | 25.6 % | 74.4 % |  |  |  |  |
| **Have CMPA complaints decreased during the COVID-19 pandemic process?** | | | | |  |  |
| No Answer | No | Yes |  |  |  |  |
| 2.4 % | 62.6 % | 35.0 % |  |  |  |  |
| **Have there been any changes in the individuals who look after the baby during the COVID-19 pandemic?** | | | | | | |
| No Answer | No | Yes |  |  |  |  |
| 0.4 % | 92.3 % | 7.3 % |  |  |  |  |
| **Have they been vaccinated during the COVID 19 pandemic?** | | | | |  |  |
| No Answer | No | Yes |  |  |  |  |
| 0.8 % | 7.3 % | 91.9 % |  |  |  |  |
