# Supplementary material for: Factors affecting formula compliance of infants with IgE mediated cow's milk protein allergy during the pandemic
Source: Front Allergy. 2023 May 5;4:1017155. doi: 10.3389/falgy.2023.1017155 (PMC10198129; doi:10.3389/falgy.2023.1017155)
Supplement: Supplementary file 2 [file Table2.docx]

**Supplementary Table 2.** Factors affecting formula-based adherence to CMPA treatment survey answer frequencies.

| **Time of birth 1) ≤36 weeks 2) 37-42 weeks 3) ≥43 weeks** | | | | | | |
| --- | --- | --- | --- | --- | --- | --- |
| 1 = 25 (10.2) | 2 = 217 (88.2) | 3 = 0 | Missing = 4 (1.6) |  |  |  |
| **Birth type 1) Vaginal delivery 2) Cesarean section** | | | | | | |
| 1 = 68 (27.6) | 2 = 172 (69.6) | Missing = 6 (2.4) |  |  |  |  |
| **Diet 1) Breast milk+Formula 2) Formula 3) Formula+Complementary foods 4) Breast milk+Formula+Complementary foods** | | | | | | |
| 1 = 18 (7.3) | 2 = 10 (4.1) | 3 = 90 (36.6) | 4 = 105 (42.7) | Missing = 23 (9.3) |  |  |
| **Is sweetener added to the formula? 1) No 2) Yes** | | | | | | |
| 1 = 139 (56.5) | 2 = 95 (37.5) | Missing = 12 (4.9) |  |  |  |  |
| **If yes, what? 1)Vanilla 2)Grape Molasses 3)Jam 4)Honey 5)Other** | | | | | | |
| 1 = 48 (33.8) | 2 = 69 (48.6) | 3 = 5 (3.5) | 4 = 3 (2.1) | 5 = 17 (12.0) | Missing = 0 |  |
| **Is the baby taking probiotics? 1) No 2) Yes** | | | | | | |
| 1 = 201 (81.7) | 2 = 33 (13.4) | Missing = 12 (4.9) |  |  |  |  |
| **Additional illness 1) Atopic Dermatitis&Eczema 2) Anemia 3) Reflux 4) Epilepsy 5) Asthma 0) None** | | | | | | |
| 1 = 9 (50.0) | 2 = 2 (11.1) | 3 = 2 (11.1) | 4 = 2 (11.1) | 5 = 3 (16.7) | 0 = 209 (85.0) |  |
| **Maternal atopy 1) no 2) yes** | | | | | | |
| 1 = 172 (69.9) | 2 = 73 (29.7) | Missing = 1 (0.4) |  |  |  |  |
| **Father atopy 1) no 2) yes** | | | | | | |
| 1 = 187 (76.0) | 2 = 56 (22.8) | Missing = 3 (1.2) |  |  |  |  |
| **Mother education status  1) Did not go to school 2) Literate 3) Primary education 4) Secondary education 5) High school 6) BS 7) MSc** | | | | | | |
| 1 = 1 (0.4) | 2 = 1 (0.4) | 3 = 15 (6.1) | 4 = 26 (10.6) | 5 = 65 (26.4) | 6 = 116 (47.2) | 7 = 22 (8.9) |
| **Father education status  1) Did not go to school 2) Literate 3) Primary education 4) Secondary education 5) High school 6) BS 7) MSc** | | | | | | |
| 1 = 1 (0.4) | 2 = 0 | 3 = 11 (4.5) | 4 = 20 (8.1) | 5 = 64 (26.0) | 6 = 129 (52.4) | 7 = 21 (8.5) |
| **Family income  1) Income more than expenses 2) Income equal to expenses 3) Income less than expenses** | | | | | | |
| 1 = 86 (35.0) | 2 = 129 (52.4) | 3 = 29 (11.8) | Missing = 2 (0.8) |  |  |  |
| **Mother's profession  1) Not working 2) Working** | | | | | | |
| 1 = 166 (67.5) | 2 = 78 (31.7) | Missing = 2 (0.8) |  |  |  |  |
| **Father’s profession  1) Not working 2) Working** | | | | | | |
| 1 = 5 (2.0) | 2 = 240 (97.6) | Missing = 1 (0.4) |  |  |  |  |
| **Mother/Father 1) Married 2) Not married** | | | | | | |
| 1 = 238 (96.7) | 2 = 1 (0.4) | Missing = 7 (2.8) |  |  |  |  |
| **Sibling(s)  1) 0 2) 1 3) 2 4) 3 5) ≥ 4** | | | | | | |
| 1 = 141 (57.3) | 2 = 65 (26.4) | 3 = 19 (7.7) | 4 = 7 (2.8) | 5 = 2 (0.8) | Missing = 12 (4.9) |  |
| **Sibling atopy  1) no 2) yes** | | | | | | |
| 1 = 103 (95.4) | 2 = 5 (4.6) |  |  |  |  |  |
| **Food allergy in family  1) no 2) yes (who?) 2.1) Sibling 2.2) Mother 2.3) Father** | | | | | | |
| 1 = 191 (77.6) | 2 = 44 (17.9) | 2.1 = 7 (2.8) | 2.2 = 6 (2.4) | 2.3 = 8 (3.3) |  |  |
| **Living environment  1) City 2) County/Town 3) Village** | | | | | | |
| 1 = 208 (84.6) | 2 = 34 (13.8) | 3 = 2 (0.8) | Missing = 2 (0.8) |  |  |  |
| **How many people live in the house? 1) 1 2) 2 3) 3 4) 4 5) ≥ 5** | | | | | | |
| 1 = 0 | 2 = 2 (0.8) | 3 = 131 (53.3) | 4 = 72 (29.3) | 5 = 41 (16.7) |  |  |
| **Does anyone aged 65 or older live at home? 1) no 2) yes (number)** | | | | | | |
| 1 = 223 (90.7) | 2 = 17 (6.9) | Missing = 6 (2.4) |  |  |  |  |
| **Who is cooking at home?  1) Mother 2) Father 3) Sibling 4) Caregiver 5) ≥ Other (please specify)** | | | | | | |
| 1 = 214 (87.0) | 2 = 8 (3.3) | 3 = 0 (51.8) | 4 = 24 (9.8) | 5 = 13 (5.3) | Missing = 23 (9.3) |  |
| **From which specialty did your child first receive the diagnosis of CMPA? 1) Family doctor 2) Child Health and Diseases Specialist 3) Pediatric Gastroenterology Specialist 4) Pediatric Allergy-Immunology Specialist 5) Other (please specify)** | | | | | | |
| 1 = 3 (1.2) | 2 = 71 (28.9) | 3 = 13 (5.3) | 4 = 157 (63.8) | 5 = 4 (1.6) |  |  |
| **Foods responsible other than cow's milk: 1) Single food allergy 2) Multiple food allergy 3) Goat milk 4) Veal 5) Eggs 6) Wheat 7) Sesame 8) Almond 9) Hazelnut 10) Walnut 11) Pistachio Nut 12) Cashew Nut 13) Peanut 14) Soy 15) Lentil 16) Chickpeas 17) Oat 18) Beans 19) Fish 20) Sea products 21) Other (please specify)** | | | | | | |
| 1 = 71 (28.9) | 2 = 127 (51.6) | 3 = 16 (6.5) | 4 = 35 (14.2) | 5 = 153 (62.2) | 6 = 27 (9.8) | 7 = 17 (6.9) |
| 8 = 14 (5.7) | 9 = 32 (13.0) | 10 = 26 (10.6) | 11 = 17 (6.9) | 12 = 6 (2.4) | 13 = 19 (7.7) | 14 = 13 (5.3) |
| 15 = 12 (4.9) | 16 = 5 (2.0) | 17 = 5 (2.0) | 18 = 5 (2.0) | 19 = 7 (2.9) | 20 = 1 (0.4) |  |
| **Elimination Diet (More than one can be marked) 1) Responsible food removed from diet 2) Cross-reactives excluded from diet (beef, other dairy, soy) 3) Responsible food diet suggested to mother 4) Cross-reactive diet suggested to mother 5) Calcium and vitamin supplementation was suggested to the mother 6) Amino acid-based formula suggested 7) Highly hydrolyzed formula suggested 8) Rice-based formula suggested 9) Baby started on complementary food 10) Other nutrient diets suggested** | | | | | | |
| 1 = 234 (95.1) | 2 = 84 (34.1) | 3 = 148 (60.2) | 4 = 45 (18.3) | 5 = 128 (52.0) | 6 = 191 (77.6) | 7 = 30 (12.2) |
| 8 = 16 (6.5) | 9 = 137 (55.7) | 10 = 23 (9.3) |  |  |  |  |
| **How many times a day does the baby breastfeed (times/day) 0) not taking 1) 1-2 2) 3-4 3) 5-6 4)>6** | | | | | | |
| 0 = 48 (19.5) | 1 = 12 (9.4) | 2 = 37 (28.9) | 3 = 30 (23.4) | 4 = 49 (38.3) | Missing = 70 (28.5) |  |
| **Who is preparing the food? 1) Mother 2) Father 3) Sibling 4) Caregiver 5) Other** | | | | | | |
| 1 = 223 (90.7) | 2 = 10 (4.1) | 3 = 0 | 4 = 14 (5.7) | 5 = 12 (4.9) | Missing = 21 (8.5) |  |
| **Has the formula been changed? 1) No 2) Yes (how many times?) 2.1) 1 time 2.2) More than 1** | | | | | | |
| 1 = 174 (70.7) | 2 = 45 (18.3) | 2.1 = 32 (13.0) | 2.2 = 12 (4.9) | Missing = 27 (11.0) |  |  |
| **Has your child had any trouble using formula? 1) No 2) Yes** | | | | | | |
| 1 = 123 (50.0) | 2 = 98 (39.8) | Missing = 25 (10.2) |  |  |  |  |
| **If yes, what is the reason? 1) Inability to provide food due to SSI problems 2) Stopping using formula because of the thought that the treatment is useless 3) Dislike the taste and smell of food, rejection of food 4) Baby stopped formula due to switching to complementary foods 5) Difficulty taking the morning or evening dose of formula 6) Concern about a decrease in breast milk** | | | | | | |
| 1 = 2 (1.7) | 2 = 1 (0.9) | 3 = 104 (88.9) | 4 = 8 (6.8) | 5 = 5 (4.3) | 6 = 3 (2.6) | Missing = 129 (52.4) |
