# Supplementary material for: Factors affecting formula compliance of infants with IgE mediated cow's milk protein allergy during the pandemic
Source: Front Allergy. 2023 May 5;4:1017155. doi: 10.3389/falgy.2023.1017155 (PMC10198129; doi:10.3389/falgy.2023.1017155)
Supplement: Supplementary file 3 [file Table3.docx]

**Supplementary Table 3.** Distribution of food allergies additional to CMPA.

| **FA distribution (in addition to CMPA)** | |
| --- | --- |
| **Protein Foods and Dairy** | **212 (86.2)** |
| Egg | 153 (62.2) |
| Veal | 35 (14.2) |
| Goat milk | 16 (6.5) |
| Fish | 7 (2.9) |
| Sea products | 1 (0.4) |
| **Tree Nuts** | **95 (38.6)** |
| Hazelnut | 32 (13.0) |
| Walnut | 26 (10.6) |
| Pistachio Nut | 17 (6.9) |
| Almond | 14 (5.7) |
| Cashew Nut | 6 (2.4) |
| **Legume and Seed** | **71 (28.9)** |
| Peanut | 19 (7.7) |
| Sesame | 17 (6.9) |
| Soy | 13 (5.3) |
| Lentil | 12 (4.9) |
| Chickpea | 5 (2.0) |
| Beans | 5 (2.0) |
| **Grain** | **32 (13.0)** |
| Wheat | 27 (9.8) |
| Oat | 5 (2.0) |

CMPA, cow-milk protein allergy

FA, food allergy
